# Platoon Merging Approach Based on Hybrid Trajectory Planning and CACC Strategies

**DOI:** 10.3390/s21082626

**Published:** 2021-04-08

**Authors:** Carlos Hidalgo, Ray Lattarulo, Carlos Flores, Joshué Pérez Rastelli

**Affiliations:** 1 Department of Automotive in the Industry and Transportation Division, Tecnalia Research & Innovation, 48160 Derio, Spain; rayalejandro.lattarulo@tecnalia.com (R.L.); joshue.perez@tecnalia.com (J.P.R.); 2 California PATH Program of the Institute of Transportation Studies, University of California Berkeley, Richmond, CA 94804, USA; carfloresp@berkeley.edu

**Keywords:** hybrid trajectory planning approach, CACC, cooperative merging

## Abstract

Currently, the increase of transport demands along with the limited capacity of the road network have increased traffic congestion in urban and highway scenarios. Technologies such as Cooperative Adaptive Cruise Control (CACC) emerge as efficient solutions. However, a higher level of cooperation among multiple vehicle platoons is needed to improve, effectively, the traffic flow. In this paper, a global solution to merge two platoons is presented. This approach combines: (i) a longitudinal controller based on a feed-back/feed-forward architecture focusing on providing CACC capacities and (ii) hybrid trajectory planning to merge platooning on straight paths. Experiments were performed using Tecnalia’s previous basis. These are the AUDRIC modular architecture for automated driving and the highly reliable simulation environment DYNACAR. A simulation test case was conducted using five vehicles, two of them executing the merging and three opening the gap to the upcoming vehicles. The results showed the good performance of both domains, longitudinal and lateral, merging multiple vehicles while ensuring safety and comfort and without propagating speed changes.

## 1. Introduction

Traffic jams in urban or highway scenarios are complex environments for implementing automated driving functionalities due to the multiple interactions with the surrounding vehicles. Traffic congestion is defined as the mutual obstruction of vehicles due to the existing interrelation among the vehicles’ speed and traffic flows [1]. On urban roads, it is common to have traffic congestion in the metropolitan areas with a high level of socio-economic development [2]. Mainly, traffic jams are triggered by situations such as lane merging, sharp cut-ins, or bottlenecks. Once triggered, these disturbances are propagated in the upstream direction as traffic shock waves. Such phenomena are explained by the fact that human drivers perform a string of unstable car-following because they tend to keep shorter inter-vehicle distances than they should, given human’s reaction capabilities [3]. This leads to an amplification of the energy of braking events coming from downstream, as it is transmitted in the upstream direction [4].

Technologies for driving assistance, such as Adaptive Cruise Control (ACC), intend to improve safety and comfort; however, traffic flow improvement is not among their design requirements [5]. It has recently been demonstrated that commercial ACC systems do not satisfy the string stability criterion. For instance, the field experiments in [6] showed how an unexpected braking situation of a platoon leader is dangerously amplified by all vehicles in the string. This demonstrates that car-following systems that rely uniquely on range measurement sensors are not sufficient to provide a close-string stable car-following. An improved version of ACC has been developed, which leverages Vehicle-to-Vehicle (V2V) communications to increase the reaction speed to disturbances, guaranteeing safety and string stability for shorter gaps at wider speed ranges. An example is Cooperative Adaptive Cruise Control (CACC), which has been demonstrated using strategies such as feed-back/feed-forward architectures with classic PD [7], fractional PD [8], or Model Predictive Control (MPC) [9]. Another example is the platoon control strategies, were two-dimensional distributed control [10,11] or adaptive optimal control [12] have been implemented. Nevertheless, CACC following a feed-back/feed-forward approach is used in most of the cases due to the string stability and the short gaps while keeping the simplicity required for embedded applications.

Car-following strategies such as CACC have proven to improve the string stability and safety by augmenting the vehicle perception with the received V2V information from forward vehicles. However, to further improve the traffic flow, additional cooperation among vehicles, as well as lateral actions are needed. Analysis regarding the market penetration of CACC [13,14] suggests that in order to mitigate the capacity reduction at merge areas, more research should be conducted in coordinating advanced merging assistance strategies with the CACC string operation. This is mainly due to the poor flexibility produced by the short-distance gaps, which lead to blockage in the lanes [13]. In addition to this, studies regarding driving safety have shown that between 240,000 to 610,000 crashes happen annually due to improper execution of lane-change and merge maneuvers, which leads to more congestion problems [15].

These maneuvers can be solved from the cooperative perspective, where two strings of vehicles coordinated among themselves, or by the nearby infrastructure, merge a unique platoon safely. In the literature, the merging of the platoon is classified into three branches: (1) on the highway from an on-ramp [16], (2) in a roundabout [17], or (3) a platoon along the highway [18].

Regarding cooperative lane-merging, numerous algorithms have been proposed, addressing the problem from a different perspective, such as: controlling the lateral motion [19], optimization of the trajectory during the maneuver [20], and maneuver negotiation through Vehicle-to-Everything (V2X) communications [21]. However, most of these approaches rely on algorithms with a high computational cost, only evaluating the behavior of the traffic without considering the dynamics of the vehicle.

One of the protocols for cooperative lane-merging was developed in the 2016 i-Game Grand Cooperative Driving Challenge (GCDC) competition [22], where two platoons on two neighboring lanes merge as one when a notification of a lane closure is received. During this process, the platoon leaders adjust their position for a smooth merge, resulting in a simultaneous pairing between both the merging platoon and the gap-opening platoon. Once the vehicles have matched their position, the lane-change process begins until they finally form one unique platoon.

This i-Game GCDC competition proposed a good protocol. However, there are still aspects to improve. This method makes extensive use of V2X messages and relies significantly on the platoon leader to coordinate the initial movements; with one merge stage, the last vehicle may slow down too much [23].

Optimal trajectory generation has been used for cooperative maneuvers [24]. Highway scenarios, with high speed and small-curvature road constraints, is where most motion planning techniques have been implemented in the last few years [25]. Some of the maneuvers studied are lane-change, obstacle avoidance, car-following, and merging. For the overtaking maneuver, trajectory generation through parametric curves and Model Predictive Control (MPC) has shown good results [26]. Other recent approaches for overtaking use a two-stage planning method to tackle the overtaking maneuver, but using pre-computed optimal curves [27]. So far, in any of these examples, a mixed approach for optimal longitudinal and lateral functionalities has not been implemented for the two platoons merging.

In this paper, a two-stage approach to manage a cooperative lane-merging maneuver is proposed. It is based on a trajectory planning algorithm using Bézier curves and MPC for path planning and a CACC strategy composed of a Feed-back/feed-forward control for string stable longitudinal car-following. The first one intends to manage the lane-change involved in the maneuver. The second is used to manage both the gap-regulation task and gap changes to perform the merging maneuver. Above these modules, a decision stage is in charge of adjusting the gaps of the platoons, processing the requests, and switching between strategies. To validate this approach, a modular architecture for automated vehicles, called AUDRIC [28], was used. Simulations were defined with five vehicles, two executing the lane-change maneuver and three opening the gap to form a unique platoon.

The rest of the paper is organized as follows: Section 2 gives an overview of the strategy implemented, based on the works [8,29]. In Section 3, the simulation frameworks and the description of the CACC and hybrid trajectory modules are presented. Section 4 shows the simulation results, and finally, in Section 5, the conclusion and future works are presented.

## 2. Platforms and Methods

### 2.1. Platoon Merging Method

**Figure 1:**
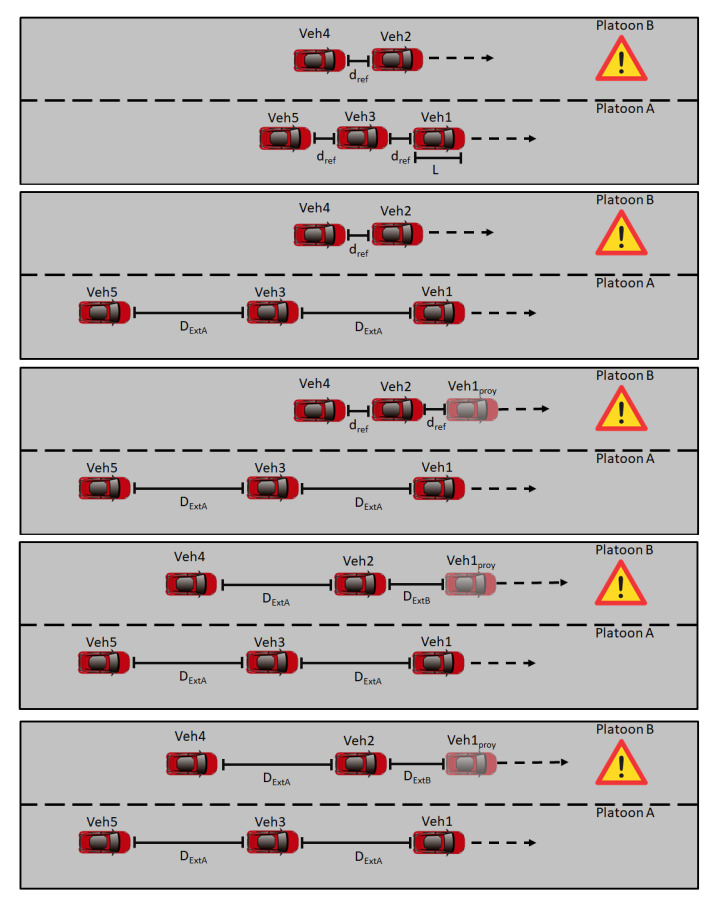
Illustration of the platoon merging maneuver as a sequence of steps, from top to bottom.

This paper focuses on the cooperative lane-merge scenario presented in Figure 1. In this scenario, Platoon B seeks to join Platoon A to avoid a problem in the route previously detected. To do so, the vehicles in Platoon A open the gap so Platoon B can safely merge, forming Platoon C. In this work, the vehicles in Platoon A are numbered as even numbers (Vehicles 2 and 4), while vehicles in Platoon B are numbered as odd numbers (Vehicles 1, 3, and 5). Only 5 vehicles were chosen to give a detailed analysis of the proposed approach; nevertheless, it can be extended to more vehicles. Based on this premise, the following considerations were taken into account:

- *L* variable stands for the vehicle length, being the same for all vehicles.
- $d_{ref}$ represents the distance reference between vehicles, explained in Section 2.2.2.
- $D_{ExtA}$ is the gap opened by Platoon A (Equation (1)), being 2 times the minimum CACC distance ($d_{std}$) defined in Section 2.2.2 plus the vehicle length (*L*). By using this relation, the vehicles in Platoon B can join around a safe position while keeping the minimum distance between vehicles.
  (1) $$D_{ExtA}=2*d_{std}+L$$
- $D_{ExtB}$ is the distance between Vehicle 2 and the projection of Vehicle 1 in the Platoon B lane.
  (2) $$D_{ExtB}=\sqrt{{\left(Pos_{x_{i-1}}-Pos_{x_{Proy}}\right)}^{2}+{\left(Pos_{y_{i-1}}-Pos_{y_{Proy}}\right)}^{2}}$$
  where $Pos_{x_{Proy}}$ and $Pos_{y_{Proy}}$ are the front projections by $D_{ExtA}/2$ in the Platoon B lane of the Platoon A vehicle that previously opened the gap.

To accomplish this goal, the combination of a trajectory planning algorithm, a car-following strategy, and a merging decision module is proposed. We used for the car-following a CACC, which ensures reliability and flexibility while executing the maneuver. Furthermore, a feed-forward plus feed-back strategy was desirable due to its simple implementation and robust results, as mentioned in previous works [7,30]. When designing a CACC system, the string stability condition is one of the most important requirements. It refers to the ability that each string vehicle has to attenuate the disturbances coming from forward vehicles [31], ensuring that the energy of exogenous disturbances decays as it propagates in an upstream direction. This requirement is satisfied if the following condition is met:

(3) $$\|\frac{X_{i}}{X_{i-1}}{\|}_{\infty }={\left\|\frac{D\left(s\right)F\left(s\right)+G_{p}\left(s\right)C\left(s\right)}{1+G_{p}\left(s\right)C\left(s\right)H\left(s\right)}\right\|}_{\infty }\leq 1;i\geq 2$$

The hybrid planning approach [29] was chosen to carry out the lane-change incorporated in the maneuver. This algorithm was based on Bézier curves and a linear MPC, to solve, safely and dynamically, the trajectory problem. This method has shown good performance, such as overtaking [32] and roundabout merging [33].

The merging decision module generates two signals: (1) the references for the gap openings and (2) the command to switch between the longitudinal gap when the lane-change occurs.

**Figure 2:**
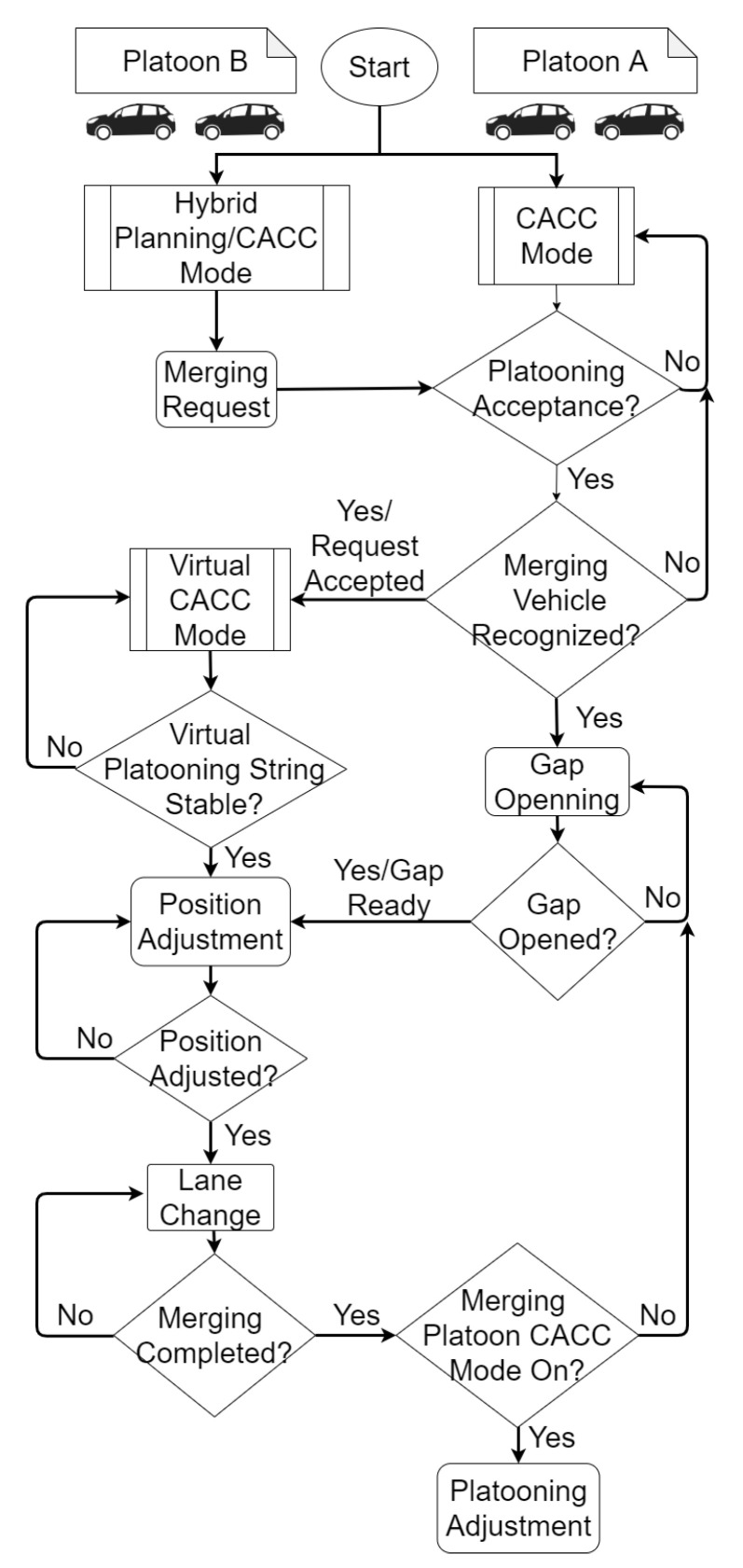
Maneuver flow state chart.

The complete algorithm is shown in the flow state chart of Figure 2. When the maneuver begins, Platoon B sends a merge request to Platoon A. If the conditions are suitable (no lane closures, accidents, etc.) and there is enough space, the request is accepted. Then, the gap is opened among the vehicles of Platoon A according to Equation (1). In parallel, Platoon B starts a virtual CACC with the projection of the Platoon A leader taking into account speed changes. The adjustment in Platoon B is based on Equation (1). Once the virtual platooning is string stable and the gap is opened, Platoon B starts positioning to begin the lane-change; during this process, the vehicles maintain the speed they currently have. Finally, when the merge is completed, Platoon B can join Platoon A, to form Platoon C. Taking into account Equations (1) and (2), the vehicles are positioned keeping the distance $d_{ref}$; therefore, the reference can be switched again to the one generated by the CACC without creating a disturbance in Platoon C. In case any vehicle of Platoon B does not want to be part of Platoon C, the gap remains open until the vehicle exits Platoon C, and then, the gap is closed once again.

### 2.2. Platform Description

**Figure 3:**
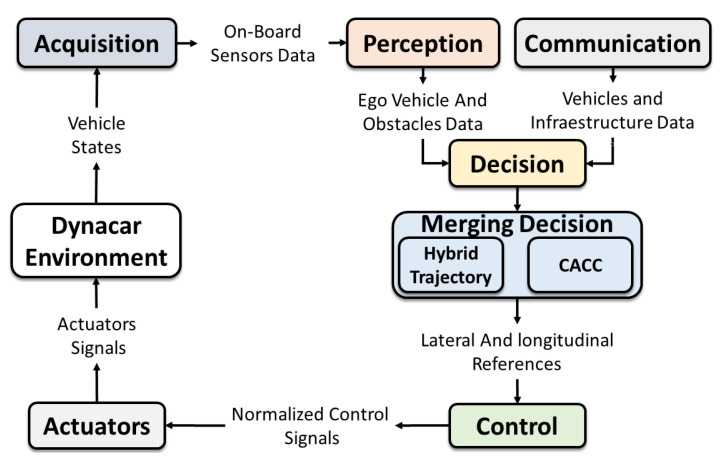
Control architecture with the merging decision block.

This section presents a description of the AUDRIC platform used to validate the merging scenario. This platform makes use of the architecture defined in [28], where the main blocks are: acquisition, perception, communication, decision, control, and actuators (Figure 3). The modeling of the vehicles was done through the Dynacarenvironment [34]. This environment counts with a multi-body model to virtually represent the vehicles [35,36], in this case a Renault Twizy 80, and a 3D interface for monitoring purpose. Since the proposed approach is not fixed to one specific simulator, the experiments can be carried out in other simulation environments such as CARLA o CarSIM.

The acquisition module is in charge of collecting all the information from the multi-body model, which allows a first representation of the current vehicle state. The perception block generates a precise ego vehicle and obstacle representation. The information from other vehicles (speed, position, heading, acceleration, dimensions, etc.) is obtained from the communication block. In this work, the simulation gives this information, considering some delays among vehicles. The decision module generates the trajectory and the actions that the vehicle has to take during the driving process. The lateral control variables (X-Y coordinates, curvature, etc.) are obtained with the hybrid approach. The longitudinal speed reference is obtained with the CACC approach. The control module is related to lateral (steering wheel) and longitudinal (throttle and brake) controllers of the vehicle. A linear double proportional controller with a feed-forward component based on the curvature is used for the lateral controller, as in [37]; and for the longitudinal, a fuzzy logic controller as in [38]. Finally, the actuation block is the one in charge of interpreting the control signals and generates values that can be used by the multi-body model.

The main contribution of this work was the development of the decision block, where the selected path and speed planners were generated to execute the platoon merging maneuver. Table 1 shows the benefits and disadvantages of both strategies, where it can be seen that both strategies complement each other, allowing both car-following and lane-change maneuvers without adding computational cost.

**Figure 4:**
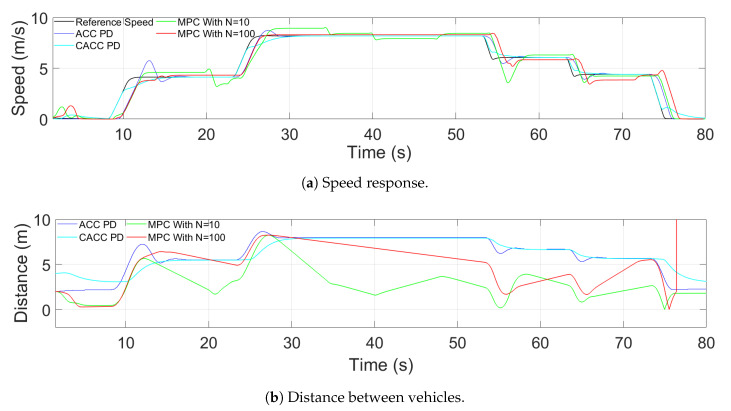
Comparison between controllers.

Regarding the longitudinal aspect of both strategies, the main difference is that the hybrid trajectory approach prioritizes safety, whereas CACC improves traffic throughput. Therefore, due to the configuration of the hybrid trajectory (Section 2.2.1), the MPC used underperformed with respect to the classic feed-back/feed-forward PD (Section 2.2.2) in car-following maneuvers. In Figure 4, this comparison is presented, where it shows that MPC could not be established in the reference, whereas the feed-back/feed-forward PD in both ACC and CACC mode could. This was mainly due to the precision obtained from the number of samples used.

**Figure 5:**
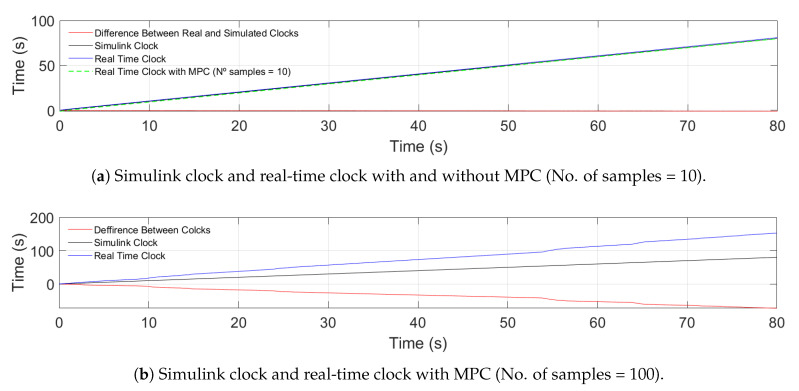
Comparative between the Simulink clock and the real-time clock with different configurations.

To improve MPC performance, the number of samples was 100 with a sample time of 0.05 s. The more predictions, the more precision there is for the measures. Figure 4 shows this, where MPC now is established in the reference. However, this change requires a higher computational cost, losing the real-time condition, as shown in Figure 5. This figure presents the Simulink clock with a real-time clock. The first one (Figure 5a) shows the performance with and without MPC of 10 samples. In both cases, the clocks tick at the same step. Meanwhile, Figure 5b contains the test with MPC using 100 samples. The difference between the clocks grows as time progresses, meaning that the system is slower.

Therefore, to guarantee a real-time performance, the feed-back/feed-forward controller is in charge of the car-following maneuver, leaving the MPC of the hybrid trajectory approach with a low number of samples to handle the lane-change maneuver.

#### 2.2.1. Hybrid Trajectory Approach

**Figure 6:**
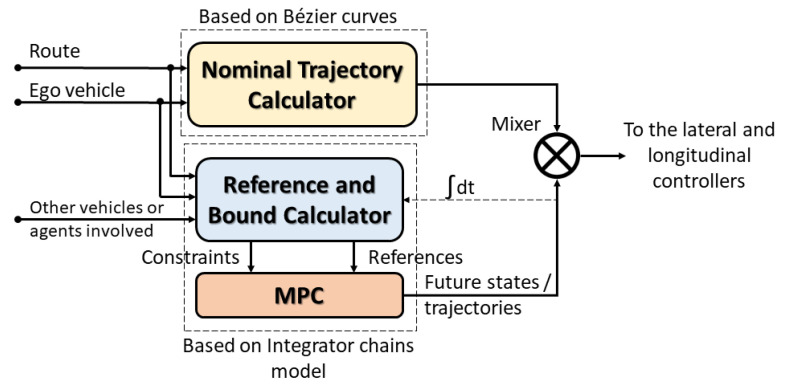
Hybrid trajectory planning block diagram.

In Figure 6, the block diagram of the hybrid trajectory approach is presented. The approach uses the information of nearby vehicles, a buffer with the route to follow, and the current ego vehicle state as inputs. This information passes through two blocks, which are the nominal trajectory calculator and the reference and bounds calculator. The first one generates the trajectory with Bézier curves, which contains a set of control variables for both lateral and longitudinal domains (lateral error, angular error, speed set-point, etc.). The references and constraints for the MPC block are calculated using the information of the previous iteration from the MPC module (previous future states’ computation). The MPC module generates the lateral and longitudinal states. Finally, the system outputs consider the minimum speed set-point between the one obtained from MPC and the one derived from the nominal trajectory calculator block. Regarding the lateral states, the lateral displacement provided by MPC is added to the lateral control error generated by the nominal trajectory calculator.

The combination of both approaches allows the vehicle to safely deal with different road components such as intersections, roundabouts, and merging, as well as maneuvers such as lane-change, overtaking, and merging. In the specific case of lane-change, the algorithm collects the information generated by MPC’s previous iteration to generate the future states of all the participants and then propagates them over the current lane and the opposite lane. In the case that no collision is detected, the lateral bounds are moved from the nominal lane to the opposite lane (merging lane), following the considerations explained in the MPC’s design.

MPC was designed following the work [39], where a linear model (point mass) with decoupled lateral and longitudinal dynamics was used. Therefore, an optimal solution with a fast response time was achieved.

The lateral model was based on a double integrator of the lateral acceleration ($a_{lat}$) component (Equation (4)).

(4) $$d_{lat}=\int \int a_{lat}\left(t\right)dt^{2}$$

This model can be presented as a linear differential equation system (Equation (5)) that involves the lateral offset ($d_{lat}$) and rate of change in the lateral offset ($v_{lat}$) as state variables and the lateral acceleration ($a_{lat}$) as the control input.

(5) $$\left[\begin{array}{c} \dot{d_{lat}}\\ \dot{v_{lat}} \end{array}\right]=\left[\begin{array}{cc} 0 & 1\\ 0 & 0 \end{array}\right]\left[\begin{array}{c} d_{lat}\\ v_{lat} \end{array}\right]+\left[\begin{array}{c} 0\\ 1 \end{array}\right]a_{lat}$$

The lateral constraints are given by the inequalities (Equation (6)):

(6) $$\begin{array}{cc} -\frac{1}{2}\left|Road_{w}\right|+\frac{1}{2}Veh_{w} & \leq d_{lat}\leq \frac{3}{2}\left|Road_{w}\right|-\frac{1}{2}Veh_{w}\\ -\left|v_{max}|\right| & \leq v_{lat}\leq \left||v_{max}|\right|\\ -\left|a_{min}|\right| & \leq a_{lat}\leq \left||a_{max}|\right| \end{array}$$

where the distance ($d_{lat}$) has dynamic constraints defined by the width of the vehicle ($Veh_{w}$) and the road ($Road_{w}$). Both acceleration ($a_{lat}$) and speed ($v_{lat}$) have static constraints defined by the maximum change of the steering wheel and the delay of the actuator.

The longitudinal model is based on a triple integrator chain of the longitudinal jerk ($J_{lon}$) component (Equation (7)).

(7) $$d_{lon}=\int \int \int J_{lon}\left(t\right)dt^{3}$$

The model can be presented as a linear differential equation system, which involves the longitudinal distance ($d_{lon}$), speed ($v_{lon}$), and acceleration ($a_{lon}$) as state variables and jerk ($J_{lon}$) as the control input, leading to the following state-space representation (Equation (8)):

(8) $$\left[\begin{array}{c} \dot{d_{lon}}\\ \dot{v_{lon}}\\ \dot{a_{lon}} \end{array}\right]=\left[\begin{array}{ccc} 0 & 1 & 0\\ 0 & 0 & 1\\ 0 & 0 & 0 \end{array}\right]\left[\begin{array}{c} d_{lon}\\ v_{lon}\\ a_{lon} \end{array}\right]+\left[\begin{array}{c} 0\\ 0\\ 1 \end{array}\right]J_{lon}$$

The variables are constrained according to (Equation (9)):

(9) $$\begin{array}{cc} 0 & \leq d_{lon}\leq d_{veh_{front}}\\ 0 & \leq v_{lon}\leq v_{limit}\\ -\left|a_{min}\right| & \leq a_{lon}\leq \left|a_{max}\right|\\ -\left|J_{min}\right| & \leq J_{lon}\leq \left|J_{max}\right| \end{array}$$

The relative distance between vehicles has variable constraints during the execution time, which goes between 0 to the maximum distance before the collision ($d_{veh_{lon}}$). The speed has a lower bound of 0 and an upper bound set by the speed limit ($v_{limit}$). The acceleration has an upper and lower bound of the maximum values allowed by the vehicle. Finally, the upper and lower bound values for the jerk are established according to passenger comfort [40].

The solution is given using a Quadratic Problem (QP) formulation minimizing the cost function (J(x(t),u(t))), which consists of the difference between the ego vehicle longitudinal speed ($v_{lon}$) and the reference generated by MPC ($v_{reflon}$) and the difference between the lateral offset ($d_{lat}$) and the lateral reference generated by MPC ($d_{reflon}$). This function is subject to the set of constraints of Equations (6) and (9) and the weighting functions *L* and *M*.

(10) $$\begin{array}{cc} \Phi (x(\cdot ),u(\cdot )) & =min\{\int_{t_{0}}^{T}L\left(J\left(x\left(t\right),u\left(t\right)\right)\right)dt\\ \; & +M(J(x(T),u(T)))\}\\ J(x(t),u(t)) & ={\left(d_{ref_{lat}}-d_{lat}\right)}^{2}+{\left(v_{ref_{lon}}-v_{lon}\right)}^{2} \end{array}$$

**Figure 7:**
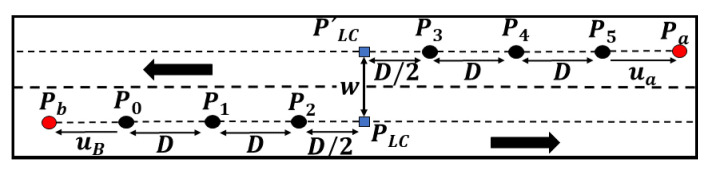
Lane-change planning example.

Bézier curves are parametric curves widely used in the automated driving field [41,42,43]. The curves have a low computation time, are easy to implement, and present geometric ($G^{n}$) and curvature continuity ($C^{n}$) [44]. The nominal trajectory generation was made using the framework developed by [45], where different road components, such as intersections, roundabouts, straight lines, and lane-changes were defined by combining Bézier curves in segments along lines and arcs. Regarding lane-change, Figure 7 shows an example of this scenario modeled according to the Bézier curve framework, where the following considerations were taken into account.

- A fifth-order Bézier curve was chosen. The control points were aligned with the lane axis to guarantee a curvature of zero at the starting and ending points. Furthermore, the point $P_{L}C$ was added to handle the maneuver in local planning.
- $u_{b}$ and $u_{a}$ are the unitary vectors for the path description and have the following relation (Equation (11)):
  (11) $$\begin{array}{cc} \vec{u_{b}} & =-\vec{u_{a}}\\ \frac{P_{b}-P_{lc}}{\left|\right|P_{b}-P_{lc}\left|\right|} & =-\frac{P_{a}-P_{lc}}{\left|\right|P_{a}-P_{lc}\left|\right|} \end{array}$$
- *w* is the width of the road, and *D* is the separation in the $u_{a}$ axis between each pair of control points (Equation (12)).
  (12) $$D=\left|\right|P_{n-1}-P_{n}{\left|\right|}_{u_{a}}$$
- The minimum value of *D* is equal to *w*, which leads to a maximum curvature in t≈20 and t≈80.

#### 2.2.2. Cooperative Adaptive Cruise Control Approach

**Figure 8:**
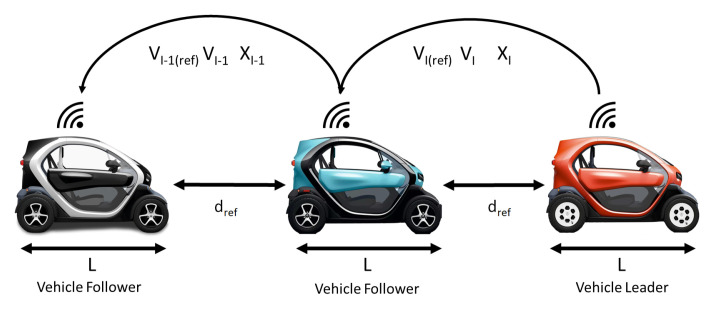
CACC scheme.

Figure 8 shows the CACC scheme used, which consisted of a homogeneous platoon of vehicles (Twizy Renault 80), with the same dynamics. The V2V communication topology employed was the Predecessor-only Following (PF) [5], where only the information (speed, position, reference speed, etc.) of the immediately preceding vehicles was used. With this structure, the string size can be increased and easily merged or split without major problems.

**Figure 9:**
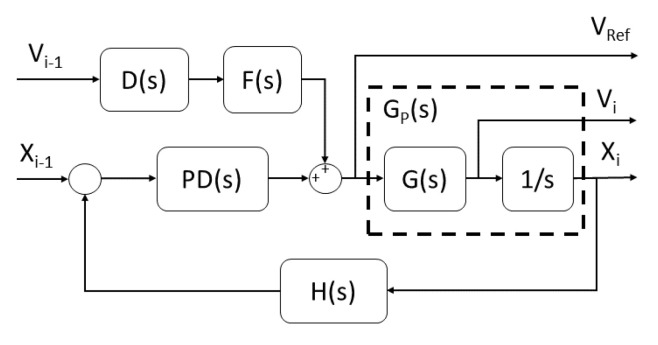
CACC block diagram.

Figure 9 presents the control architecture of the feedback controller. The G(s) block represents the second-order function used to model the vehicle response of the low-level control in charge of following the reference speed $V_{ref}$ (Equation (13)), whereas Gp(s) represents the transfer function in charge of generating the vehicle position (Equation (14)). To obtain the values for the transfer functions G(s) and Gp(s), different speed changes were introduced into the platform, and then, using the MATLAB System Identification IDE, the transfer function was obtained.

(13) $$G\left(s\right)=\frac{V_{i}}{V_{i-1}}=\frac{1.1792}{s^{2}+1.7539s+1.199}$$

(14) $$Gp\left(s\right)=\frac{G(s)}{s}=\frac{1.1792}{s*(s^{2}+1.7539s+1.199)}$$

The PD(s) block is the gap regulation block, which consists of a Proportional Derivative (PD) controller (Equation (15)), which presents a good performance with feed-forward structures [46]. The controller values were obtained according to [47], ensuring a robust system response towards loop gain variations.

(15) $$PD\left(s\right)=K_{p}+K_{d}s=0.5393+0.4103s$$

The H(s) block denotes the spacing policy. The constant time gap policy [48] (Equation (16)) was selected, where $d_{std}$ is a fixed standstill distance, $h_{d}$ is the time gap, and v(t) is the vehicle speed. This relation can be represented as Equation (17).

(16) $$d_{ref}\left(t\right)=d_{std}+h_{d}v\left(t\right)$$

(17) H(s)=1+hs

The F(s) block stands for the feed-forward filter, which was selected as Equation (18) to ensure theoretical string stability assuming no communication delay and a homogeneous platoon.

(18) $$F\left(s\right)=\frac{1}{H(s)}$$

**Figure 10:**
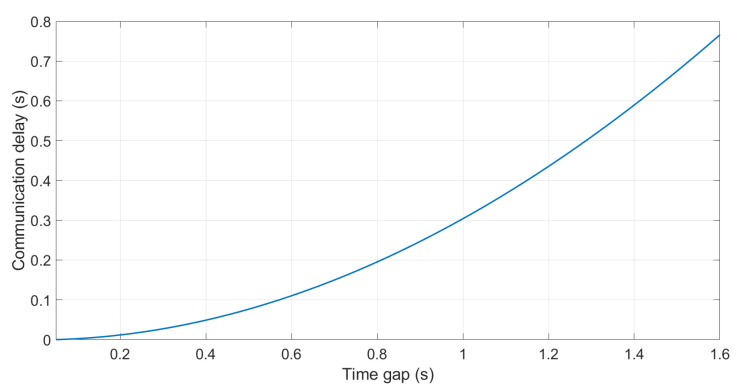
Delay vs. minimum time gap analysis.

Finally, the D(s) block represents the communication delay. This work assumed 0 (Equation (19)). However, to fully understand the system limitations, stability analyses were done, modifying the delay and observing the minimum time gap allowed that could be set while fulfilling the string stability condition. This analysis is shown in Figure 10, where a maximum time gap of 0.6 s can be employed when delays of 100 ms are present.

**Figure 11:**
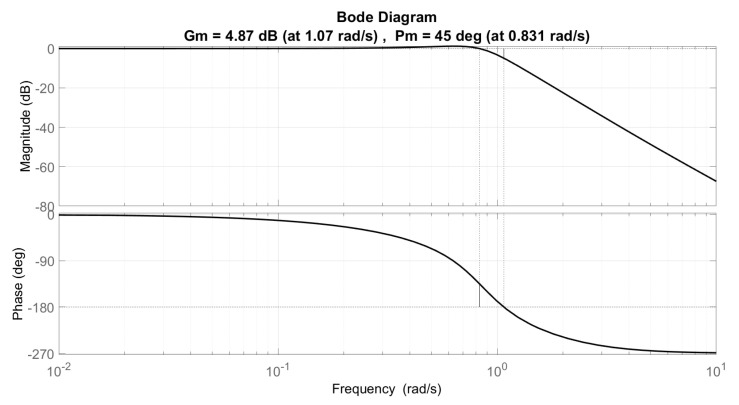
String stability frequency analysis.

Regarding the string stability criterion shown in Figure 11, we present the Bode plot of the system frequency analysis, where it can be seen that this criterion is fulfilled.

(19) $$D\left(s\right)=e^{-\theta *s}$$

A linear function of the time gap was chosen to carry out the gap opening procedure [8]. This function depends on the distance between vehicles d(t) and the ego vehicle speed (v(t)). Knowing the final distance ($d_{final}$) and speed ($v_{final}$), the desired time gap ($h_{final}$) was calculated (Equation (20)). To do so, a comfortable acceleration was applied until the vehicle distance and the ego vehicle speed yielded $h_{final}$. The higher this acceleration is, the faster the time gap will be reached. In case of Platoon A, $d_{final}$ is equal to $D_{ExtA}$, whereas, for Platoon B, $d_{final}$ corresponds to the distance formed by the virtual vehicle and the gap opened by Platoon A. Finally, $v_{final}$ is equivalent to the speed of the Platoon A leader.

(20) $$\begin{array}{cc} \; & h\left(t\right)=\frac{d\left(t\right)-d_{std}}{v(t)}\\ \; & h_{final}=\frac{d_{final}-d_{std}}{v_{final}} \end{array}$$

## 3. Results

**Figure 12:**
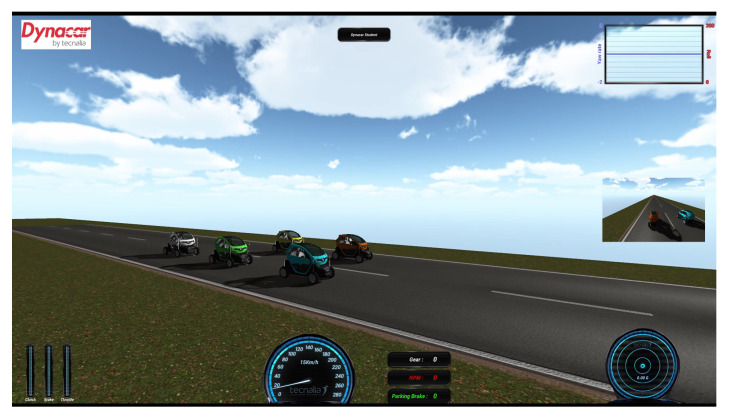
Maneuver simulation.

The simulation was performed with the Dynacar simulator environment. Five vehicles were represented, two in the left lane forming Platoon B and three in the right lane forming Platoon A (Figure 12). During the merging maneuver, a constant speed of 15 km/h was established for the leader, simulating a low-speed platoon in urban scenarios. Additionally, a constant time gap of 0.6 s and a standstill distance of 3 m were selected for the CACC maneuver.

**Figure 13:**
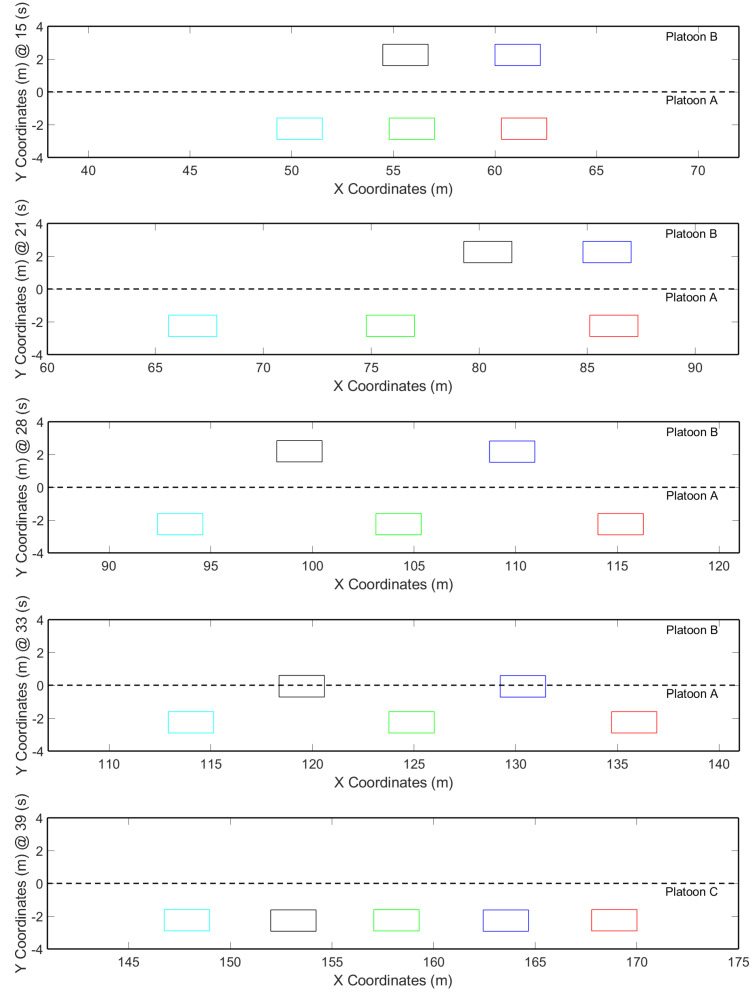
Vehicle position during the maneuver.

Figure 13 shows the position of the vehicles at each time interval, where a smooth change in the position from Platoon B to Platoon A can be appreciated. A new Platoon C is formed.

**Figure 14:**
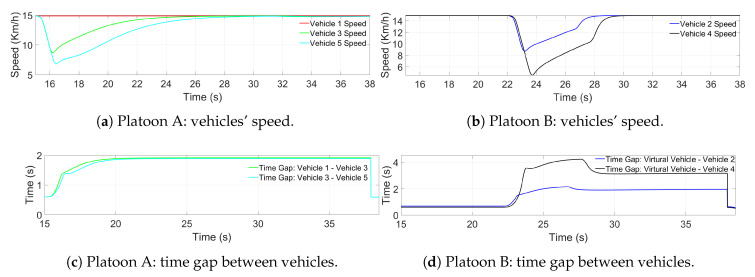
Platoon longitudinal performance during merging.

Figure 14 shows the longitudinal performance of both platoons during the merging. Figure 14a shows the vehicle speed of Platoon A, whereas Figure 14b presents the vehicle speed of Platoon B. The time gaps between vehicles during the merging process are depicted in Figure 14c,d, corresponding to Platoon A and Platoon B, respectively.

**Figure 15:**
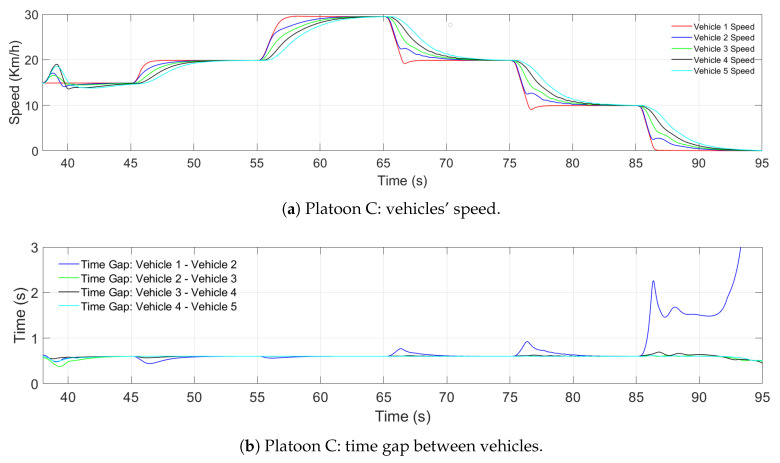
Platoon C longitudinal performance.

Figure 15 shows the longitudinal performance of Platoon C, from the moment the vehicles finished merging. Figure 15a presents the vehicle speed and Figure 15 the time gap among the vehicles.

**Figure 16:**
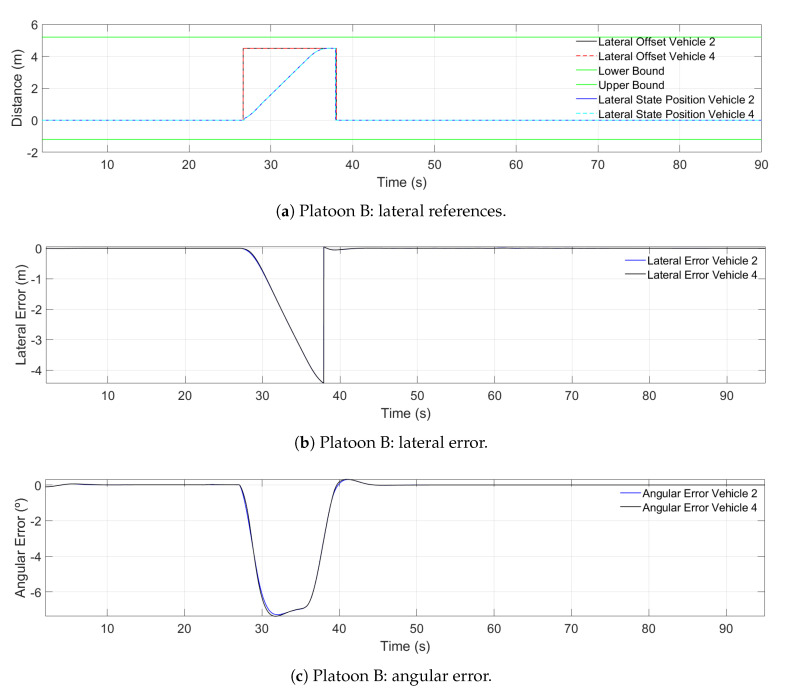
Platoon B: lateral performance during merging.

In Figure 16, the lateral performance of Platoon B during the merging maneuver is presented. Figure 16a shows the lateral references generated by the hybrid trajectory planning, and Figure 16b,c shows the lateral error and angular error.

## 4. Discussion

In Figure 13 at Second 15, the beginning of the process can be seen by sending the merge request. Around Second 21, Platoon A finishes opening the gap and sends the gap confirmation. This action ensures that only the vehicle in Platoon B can merge when safe conditions are present. The first of these conditions is a safe distance among the vehicles, which is achieved with Equation (1). Then, at Second 28, Platoon B finishes adjusting its position according to the gap opened by Platoon A. At Second 33, Platoon B executes the lane-change, and finally, at Second 39, both platoons are merged into one.

Concerning the longitudinal performance, Figure 14a shows Second 15 when Platoon A behind Platoon B decelerates to open the gap. Figure 14b shows that at around Second 23, Platoon B decelerates using as a reference the projection of the vehicle leader of Platoon A and positioning parallel to the space opened by Platoon A. At Second 28, the longitudinal reference is switched to that generated by MPC, to execute the lane-change. At Second 39, Platoon B finishes the lane-change and switches back to the CACC longitudinal reference, adjusting the position in the newly formed Platoon C.

Regarding the time gap, it can be observed in Figure 14c, that the gap among vehicles opened up until $h_{Final}$ was reached without amplification. Figure 14d shows the vehicles in Platoon B, incrementing the gap between them and the virtual vehicle until the lane-change position is achieved. Once the lane-change has finished, the vehicles adopted as references the vehicles in front of them.

Finally, once the merge was complete, Figure 15a shows that between Seconds 39 and 45, the vehicles closed the gaps. Nevertheless, the merge was completed successfully, and the fact that the vehicles needed to adjust their positioning indicates they did not merge into the expected position. After the vehicles stabilized in the lane, different speed changes were introduced to show the correct performance of the CACC, not amplifying the changes while maintaining the references. In Figure 15, in the last 10 s, when a sudden brake is implemented in Vehicle 1, the actual time gap between Vehicle 1 and Vehicle 2 goes to infinity. The main reason is due to the relation used to obtain the value; however, this behavior does not affect the performance of the platoon, as can be seen in the rest of the vehicles, where the progression from their current speed to zero is achieved smoothly.

Regarding the lateral performance of Platoon B during the merging, in Figure 16a at Second 26, the lane-change maneuver begins, so the boundaries are modified to allow the change in the reference. Once Platoon B is in the other lane, the reference is set to 0 m and starts using the trajectory followed by Platoon A. Additionally, it can be observed that, since both vehicles perform the maneuver at the same time and have equal dynamics, there is no difference in the lance change execution. Figure 16b,c shows the lateral error and angular error. The peaks values are 4.5 m and $7.27^{\circ }$, respectively. Moreover, the progression of both variables shows the comfort of the maneuver. Nevertheless, due to ensuring comfort and safety in the maneuver, the lane-change process takes 12 s, a value that is almost an average (1 s to 13.33 s [49]).

Overall, the approach implemented was considered a good solution, ensuring safety without amplifying speed changes in the process. However, the time it took to execute the maneuvers can be improved, as well as the positioning of the vehicles, once the lane-change is finished. In future works, these aspects will be addressed, as well as a real implementation using Renault Twizy 80 vehicles, since the simulator used allows a reliable vehicle representation.

## 5. Conclusions

This paper presents an approach to overcome the cooperative lane-merging maneuver. The combination of a trajectory algorithm based on hybrid planning (Bézier curves and MPC) to deal with the lane-change with a car-following algorithm composed of a feed-back/feed-forward controller was proposed.

The simulations of two platoons were carried out with the AUDRIC/Dynacar environment, showing the good performance of the strategy. Platoon B safely merged into the other lane, by positioning parallel to the gap opened by Platoon A and executing a smooth lane-change without any perturbations. This decentralized method required low computational cost without depending on the platoon’s leaders.

In the approach, five vehicles were used, but it is not limited to that amount, since no amplification in the speed changes is present. However, the proposed approach can be improved in the future, reducing the time in which the lane-change is executed and the positioning of the vehicles once the lane-change is finished, so no adjustments would be necessary, especially if more demanding scenarios were present.

Concluding the work, the approach sets the basis for future implementations considering other merging-related scenarios. It possesses a planning capable of taking into account different road components, as well as executing different maneuvers, alongside a CACC algorithm that allows the manipulation of the vehicle gaps without propagating speed changes. Therefore, future works include: (i) setting a minimum speed during the gap-opening process to avoid the possible stopping of the last vehicle if considering a large number of vehicles per platoon, (ii) algorithm adaptation to perform CACC in curved scenarios such as roundabouts, (iii) improving the car-following strategy to allow the merging of vehicles with different dynamics, and (iv) executing the maneuver in a real scenario with V2X communications.

## Figures and Tables

**Table 1:** Benefits and downside of CACC and hybrid trajectory.

	Benefits	Disadvantage
CACC	Allows short gaps between vehicles	No actions/usage in the lateral component
	Ease of handling the gap for cut-in, cut-out maneuvers	
	Easy implementation	Difficult lateral displacement
	Low computational cost	
	String stability guaranteed	
Hybrid trajectory	Actions in both longitudinal and lateral components	Average to high computational cost
	Allows lateral maneuvers such as lane-change	
	Collision avoidance through projections of future states	No short gaps allowed
	Passenger comfort guaranteed	
	Easy-to-medium implementation	

## Data Availability

Not applicable.
